# Study of Fresh and Hardening Process Properties of Gypsum with Three Different PCM Inclusion Methods

**DOI:** 10.3390/ma8105324

**Published:** 2015-09-24

**Authors:** Susana Serrano, Camila Barreneche, Antonia Navarro, Laia Haurie, A. Inés Fernandez, Luisa F. Cabeza

**Affiliations:** 1 GREA Innovació Concurrent, Edifici CREA, University of Lleida, C/Pere de Cabrera S/N, Lleida 25001, Spain; sserrano@diei.udl.cat (S.S.); cbarreneche@diei.udl.cat (C.B.); 2 Department of Materials Science & Metallurgical Engineering, University of Barcelona, Barcelona 08028, Spain; ana_inesfernandez@ub.edu; 3 Grup Interdisciplinar de Ciència i Tecnologia en Edificació (GICITED), Departament Construccions arquitectòniques II, Universitat Politècnica de Catalunya, Barcelona 08034, Spain; antonia.navarro@upc.edu (A.N.); laia.haurie@upc.edu (L.H.)

**Keywords:** phase change material (PCM), gypsum, fresh state, coating, building

## Abstract

Gypsum has two important states (fresh and hardened states), and the addition of phase change materials (PCM) can vary the properties of the material. Many authors have extensively studied properties in the hardened state; however, the variation of fresh state properties due to the addition of Micronal® DS 5001 X PCM into gypsum has been the object of few investigations. Properties in fresh state define the workability, setting time, adherence and shrinkage, and, therefore the possibility of implementing the material in building walls. The aim of the study is to analyze, compare and evaluate the variability of fresh state properties after the inclusion of 10% PCM. PCM are added into a common gypsum matrix by three different methods: adding microencapsulated PCM, making a suspension of PCM/water, and incorporating PCM through a vacuum impregnation method. Results demonstrate that the inclusion of PCM change completely the water required by the gypsum to achieve good workability, especially the formulation containing Micronal® DS 5001 X: the water required is higher, the retraction is lower (50% less) due to the organic nature of the PCM with high elasticity and, the adherence is reduced (up to 45%) due to the difference between the porosity of the different surfaces as well as the surface tension difference.

## 1. Introduction 

The International Energy Agency (IEA) defines the building envelope as “the boundary between the conditioned interior of a building and the outdoors”. Thereby, the composition of all building parts is critical in determining how much energy is required for conditioning. Space heating and cooling account for one-third of all energy consumption, therefore the building envelope cannot be underestimated [1,2]. The implementation of passive systems with PCM in the building envelope are used to store/release heat in order to reduce the energy consumption in building sector [3,4]. A wall with PCM can store high amounts of heat using solar radiation due to its high thermal mass and the temperature fluctuation inside the building can be reduced [5].

In the last decades, a lot of scientific studies where PCM are implemented in the building envelope have been published [6,7,8,9]. In this publication, properties in hardened state of gypsum, such as mechanical and thermal properties, are extensively studied. In addition, the highest amount of PCM ever incorporated in a gypsum board was achieved (45% in weight) in [10,11] and the results show that the new gypsum with 45% of Micronal® (BASF, Ludwigshafen, Germany) DS 5001 X PCM stores five times more energy per mass unit than a thermal brick wall, 9.5 times more than a brick wall, and almost three times more than a common gypsum board under the same test conditions. However, there is a gap on the study of fresh state properties, which affect considerably the production process and the application of the material in the building site. Organic substances like molasses can be added into gypsum in order to control the hardening process [12]. It is expected that fresh state properties of gypsum will change with the addition of PCM, but the extent of this variation is not known. There are some studies which evaluate the variation of properties due to the addition of PCM in different materials. Cunha *et al.* 2013 [13], for example, studies the influence of adding different proportions (0%, 10%, 20% and 30%) of two types of PCM in the fresh and hardened properties of gypsum mortars.

In the present investigation, 10% of phase change material (PCM) is added into a common gypsum matrix (E-35) by three different methods: adding microencapsulated PCM (Micronal® DS 5001 X), making a suspension of the PCM in water, and incorporating the PCM through a vacuum impregnation method. RT-21 paraffin wax PCM (Rubitherm, Berlin, Germany) was used in the suspension and impregnation formula. Gypsum has two important states during the implementation process: fresh and hardened states. Fresh state properties of gypsum include properties in fresh state (in liquid form) and during the hardening process so properties in fresh state define the workability of the material: setting times, shrinkage, consistency, and adherence. The study of fresh state properties is mandatory in order to implement the material into the building. The aim of the research presented is to analyze, compare and evaluate the variability of gypsum properties during fresh state after the addition of 10% of PCM by three different methods as a novelty in the research area of PCM added into gypsum. 

## 2. Materials and Method 

### 2.1. Materials

Hemihydrate gypsum with high purity and minimum flexural resistance of 3.5 N/mm^2^ (according to UNE-EN 13279:2006 [14]), codified as E-35 and classified as Euroclass A1 (without fire contribution) according to 89/106/CEE Directive, is used in this experimentation as the matrix of all materials formulated. It is commercialized by PlacoSaintGobain and supplied by Joaquim Closas Sabadell.

The inclusion of the PCM into the gypsum was performed by three different methods. The amount of PCM used in this experimentation is 10% in weight, regardless the method used. The methods are described below:

- Addition of microencapsulated PCM powder Micronal® DS 5001 X from BASF (Ludwigshafen, Germany). Gypsum and microencapsulated PCM are firstly mixed and afterwards the kneading water is added to obtain the material.
- Suspension of the required water to hydrate the gypsum and paraffin RT-21 from Rubitherm. Then, this suspension is mixed with the powder gypsum.
- Vacuum impregnation of RT-21 paraffin into hardened gypsum samples.

RT-21 has 21 °C melting point and 155 kJ/kg melting enthalpy. Micronal® DS 5001 X has a melting point around 26 °C and its melting enthalpy is 110 kJ/kg. Distilled water is used to hydrate the gypsum and provide workability as UNE-EN 13279-2 specifies [15]. 

A reference (gypsum without PCM) is used in order to evaluate the variation of the results when PCM is added into the formulation by the three different methods. Furthermore, the consistency of the final material (gypsum and gypsum with PCM) used in the experimentation was fluid to allow the workability of the material during the experimentation. 

The nomenclature and sample formulations used during the experimentation are detailed in Table 1.

**Table 1:** Nomenclature and sample formulations (percentages in weight). PCM: phase change materials.

Type	Sample	Gypsum (%)	PCM (%)	Water (%)
Gypsum without PCM	REF	60	0	40
Microencapsulated PCM + Gypsum	M	45.5	10	44.5
Suspension RT-21 + Gypsum	S	50	10	40
Impregnation RT-21 + Gypsum	I	60	10	40

### 2.2. Methodology

#### 2.2.1. Consistency

**Figure 1:**
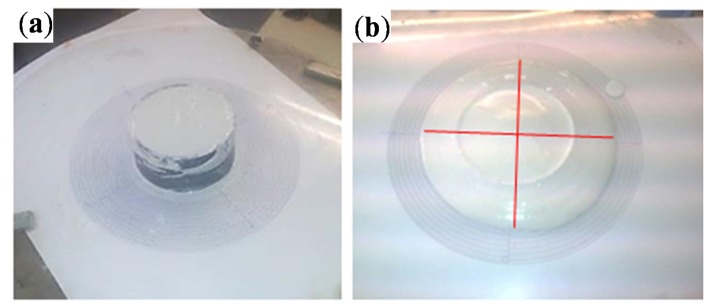
(**a**) Starting the flow value test; (**b**) after the run-off of the gypsum.

The workability is a key property which must be controlled during the implementation process of the gypsum in the wall. By achieving fluid consistency, the workability can be guaranteed. This property is directly related with the water/gypsum content (which can be calculated following the Equation (1) UNE-EN 13279-2 [15], where *R* is the consistency, *m*_1_ is the water content in grams (g) and *m*_2_ is the gypsum content (REF and type I), or gypsum and PCM content (types M and S) in grams. In order to obtain fluid consistency and, therefore, a good workability, the flow value (which is calculated as the average of perpendicular diameters as Figure 1 shows) must be between 150–210 mm. Gypsum must be mixed during 3 min following the steps described in [15] and tested immediately after the mixing process in rubber cone-shape molds of 40 mm of height and inner diameters of 65 mm and 75 mm.

(1) $$R=\frac{m_{1}}{m_{2}}$$

#### 2.2.2. Setting Times by Vicat Method

**Figure 2:**
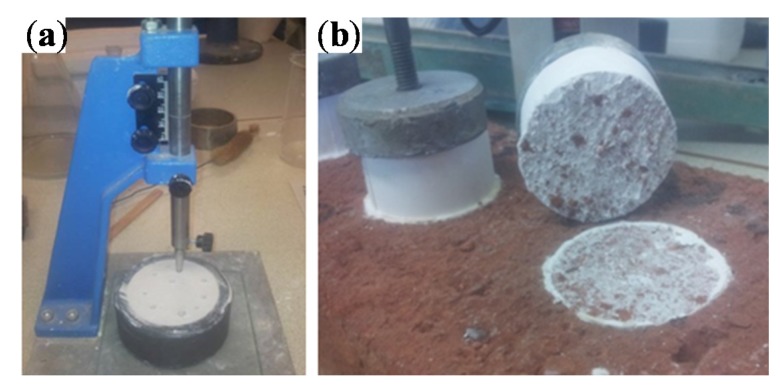
(**a**) Vicat needle; (**b**) Adherence test.

Vicat needle is used (see Figure 2a) to determine the setting times of gypsums by the penetration of the needle inside the material during the hardening process following UNE-EN 13279-2 [15]. Samples are performed in rubber cone-shape molds of 40 mm of height and inner diameters of 65 and 75 mm. Setting times can be calculated with Equation (2) where *T*_i_ is the setting time of the gypsum in min, *t*_1_ is the time in minutes when Vicat Needle achieves 22 ± 2 mm of depth and *t*_0_ is the time in minutes when water and gypsum are in contact. This test determines the initial time of hardening process and finalizes when the needle sinks 22 ± 2 mm of depth, which means that the hardening process is finalized.

(2) $$T_{\text{i}}=t_{\text{1}}-t_{\text{2}}$$

#### 2.2.3. Adherence

Figure 2b shows KN-10 of Neurtek (Eibar, Spain) where the adherence of gypsum is tested. The adherence is tested and calculated following Equation (3) where *R*_u_ is the adherence force, *F*_u_ in Newtons is the load and *A* in mm^2^ is the area of the sample tested as the standard UNE-EN 13279-2 [15] describes. The test consists on the application of a perpendicular traction force between gypsum samples and ceramic or concrete surfaces fixed by dual-component resins. Three samples per type of 50 mm of diameter and 20 mm of thickness (Figure 2) are analyzed. Samples must be kept in laboratory conditions during 7 days before testing.

(3) $$R_{\text{u}}=\frac{F_{\text{u}}}{A}$$

#### 2.2.4. Dimensional Stability

**Figure 3:**
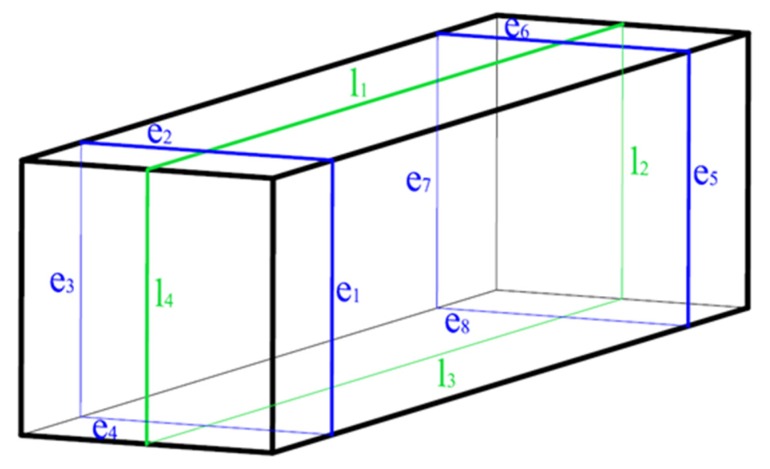
Measured points of samples in dimensional stability test (l corresponds to longitudinal dimensions, e corresponds to transversal dimensions).

Longitudinal, transversal and volumetric shrinkage appear during the hardening process of gypsum. Thus, changes in length and volume of 160 mm × 40 mm × 40 mm samples are controlled. The thickness of the sample is calculated as the average of 9 measures at different points, and the length is calculated as the average of 4 measures (from l_1_ to l_4_) as Figure 3 illustrates. 

## 3. Results

The gypsum consistency as a fresh state property, and setting times and adherence as properties during the hardening process of the three types of gypsum with PCM under study are tested. The results obtained are evaluated and compared with the reference, which is gypsum without PCM.

The results of flow value and water/gypsum content obtained are listed in Table 2. In this test, water/gypsum contents were fixed in order to obtain flow values between 150–210 mm, and thus, fluid consistency, which is the final goal of the test. It can be seen that I type has the same behavior than REF because the PCM is added into the gypsum after the hardening and curing process, so the relation water/gypsum is 0.7 in both cases. Fluid consistency can be achieved by the addition of 10% of microencapsulated PCM, 50% of gypsum and 40% of water with a water/gypsum relation of 0.8. Therefore, a suitable workability is achieved in type M with a higher water/gypsum (*w*/*s*) ratio. However, the flow value of type S has not been calculated because it hardens rapidly (see setting times listed in Table 3). Paraffin acts as inert filler and hence it does not interact in the hydration process of gypsum. Fluid consistency and, therefore, good workability of type S cannot be guaranteed.

Setting times calculated by Vicat needle test are listed in Table 3. The addition of PCM before the hardening in fresh state shortens the hardening process in all cases. Type M hardens 13 min faster than REF, which hardens in 39 min. The addition of suspended PCM (type S) accelerates the hardening process drastically, taking only 8 min. Setting times are not modified in type I.

**Figure 4:**
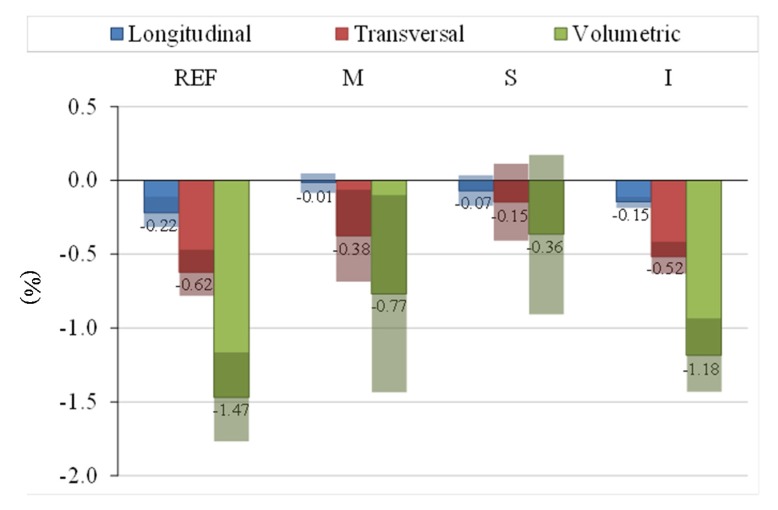
Dimensional variations and uncertainties of measures in %.

In general, the addition of PCM reduces the variation of volume (volume increase) during the hardening process if results are compared with the reference as the average values in Figure 4 demonstrate. Particularly, retraction is strongly reduced in types M and S. However, results are not homogeneous and there are big uncertainties in these measures. 

**Figure 5:**
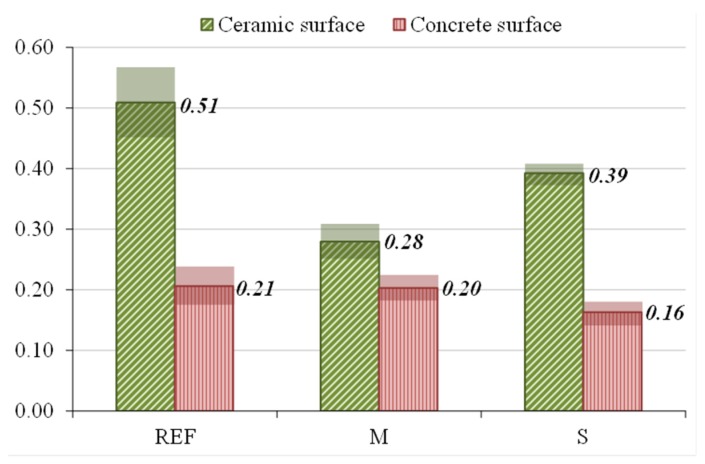
Average adherence and uncertainties of measures in ceramic and concrete surfaces in N/mm^2^.

In order to evaluate the adherence of the three types of gypsum with PCM, two types of surfaces with different porosities are used: ceramic (15% of porosity) and concrete (10% of porosity) surfaces. In general, the adherence of gypsum (with or without PCM) is better in a ceramic surface due to its higher porosity as Figure 5 confirms. The adherence of type I cannot be calculated because the PCM is added after gypsum hardening.

**Table 2:** Flow values and consistency.

Flow Values	REF	M	S	I
Gypsum (%)	60	45.5	50	60
Water (%)	40	44.5	40	40
PCM (%)	0	10	10	0
Diameter 1 (mm)	164	167	-	164
Diameter 2 (mm)	164	169	-	164
Flow value (mm)	164	168	-	164
Water/Gypsum (gypsum + PCM)	0.7	0.8	0.7	0.7

**Table 3:** Setting times by Vicat needle test.

Sample	Setting Times (min)
REF	39
M	26
S	8
I	39

Adherence to the ceramic surface is reduced by 45% for the gypsum incorporating 10% of microencapsulated PCM (type M), and 35% by the addition of suspended PCM (type S). 

In concrete surface, the adherence of REF and M are 0.20 N/mm^2^ in both cases, however, with the addition of suspended PCM the adherence is reduced by 15%, being 0.17 N/mm^2^ (type S).

## 4. Discussion

In order to make feasible the use of PCM in building materials the effect on a broad range of properties has to be evaluated. Most of the articles study the thermal behavior of the material containing PCM, but little references have been found about the influence of PCM on fresh state properties of gypsum. Consistency and setting time will determine if the gypsum could be applied *in situ*. Reduction of adherence to conventional surfaces, such as bricks or concrete, would also limit the use of the gypsum formulations. 

The results show strong variations of properties if PCM is added into gypsum and, as expected, the inclusion method also affect fresh state properties. Both PCM used accelerate setting times, especially RT-21 which reduces by 80% the setting time. However, it must be taken into account that the ratio water/gypsum is lower because a water percentage is replaced by PCM. This behavior can be a disadvantage if the gypsum has to be applied *in situ* because workers will not have enough time to place the material in the wall. Adding set time retarders can be a possible solution for *in situ* applications. On the other hand, PCM could be added in prefabricated gypsum boards, where the production process can be controlled, moreover, PCM could be added by suspension or vacuum impregnation process. 

As expected, the required water to hydrate the gypsum is higher with microencapsulated PCM because microcapsules act as an aggregate with high specific surface. The addition of microencapsulated PCM into the gypsum reduces the expansion (longitudinal, transversal and volumetric) in all cases. Taking into account average results, volumetric variations are specially reduced by the addition of 10% of microencapsulated PCM and RT-21 by suspension method into the gypsum. Microencapsulated PCM acts as strain scattering during the hardening process and for this reason volumetric expansion is reduced. However, measures are very diffuse with big uncertainties.

The adherence is also strongly modified if PCM is added into gypsum. The adherence to a ceramic surface is reduced by 45% and 35% if microencapsulated PCM and paraffin are added into gypsum, respectively. In a concrete surface, this reduction is only noticed with paraffin, which reduces by 15% the adherence. This behavior is due to the differences in porosities of both surfaces used: the ceramic brick has higher porosity (15%) than the concrete (10%). Moreover, differences on surface tension could also explain the adherence reduction with comparison with plain gypsum. Otherwise, for impregnated samples it is useless to calculate this parameter as the PCM is impregnated on hardened samples.

## 5. Conclusions

The inclusion of PCM significantly modifies the fresh properties of gypsum. The high specific surface of microencapsulated PCM (Micronal® DS 5001 X) increases the water ration required for gypsum hydration. PCM reduce the volume expansion of gypsum probably thanks to their high elasticity. Adherence of the PCM/gypsum formulations is highly dependent on the porosity of the receiving surface and decreases on surfaces with lower porosity. Differences on surface tension could also explain the adherence reduction with comparison with plain gypsum. 

The type of PCM as well as the inclusion method selected gives rise to different kind of gypsum products. Gypsum with 10% of microencapsulated PCM could be supplied for *in situ* application, while the authors would not recommend the suspension method for this application. 

Nevertheless, vacuum impregnation and suspension would gain interest to introduce PCM into prefabricated gypsum boards. Furthermore, the vacuum impregnation process could allow the addition of higher PCM loadings to enhance the thermal performance of the material.
